# Phase Transition and Coefficients of Thermal Expansion in Al_2−*x*_In*_x_*W_3_O_12_ (0.2 ≤ *x* ≤ 1)

**DOI:** 10.3390/ma14144021

**Published:** 2021-07-18

**Authors:** Andrés Esteban Cerón Cortés, Anja Dosen, Victoria L. Blair, Michel B. Johnson, Mary Anne White, Bojan A. Marinkovic

**Affiliations:** 1 Department of Chemical and Materials Engineering, Pontifical Catholic University of Rio de Janeiro (PUC-Rio), Rio de Janeiro 22453-900, Brazil; ceron@aluno.puc-rio.br (A.E.C.C.); adosen@puc-rio.br (A.D.); 2 DEVCOM Army Research Laboratory, 6300 Rodman Rd. APG, Adelphi, MD 21005, USA; victoria.l.blair3.civ@mail.mil; 3 Clean Technologies Research Institute, Dalhousie University, Halifax, NS B3H 4R2, Canada; michel.johnson@Dal.Ca (M.B.J.); mary.anne.white@dal.ca (M.A.W.); 4 Department of Chemistry, Dalhousie University, Halifax, NS B3H 4R2, Canada

**Keywords:** low thermal expansion, AlInW_3_O_12_, high-temperature XRPD, dilatometry, DSC

## Abstract

Materials from the*A*_2_*M*_3_O_12_ family are known for their extensive chemical versatility while preserving the polyhedral-corner-shared orthorhombic crystal system, as well as for their consequent unusual thermal expansion, varying from negative and near-zero to slightly positive. The rarest are near-zero thermal expansion materials, which are of paramount importance in thermal shock resistance applications. Ceramic materials with chemistry Al_2−*x*_In*_x_*W_3_O_12_ (*x* = 0.2–1.0) were synthesized using a modified reverse-strike co-precipitation method and prepared into solid specimens using traditional ceramic sintering. The resulting materials were characterized by X-ray powder diffraction (ambient and in situ high temperatures), differential scanning calorimetry and dilatometry to delineate thermal expansion, phase transitions and crystal structures. It was found that the *x* = 0.2 composition had the lowest thermal expansion, 1.88 × 10^−6^ K^−1^, which was still higher than the end member Al_2_W_3_O_12_ for the chemical series. Furthermore, the AlInW_3_O_12_ was monoclinic phase at room temperature and transformed to the orthorhombic form at ca. 200 °C, in contrast with previous reports. Interestingly, the *x* = 0.2, *x* = 0.4 and *x* = 0.7 materials did not exhibit the expected orthorhombic-to-monoclinic phase transition as observed for the other compositions, and hence did not follow the expected Vegard-like relationship associated with the electronegativity rule. Overall, compositions within the Al_2−*x*_In_*x*_W_3_O_12_ family should not be considered candidates for high thermal shock applications that would require near-zero thermal expansion properties.

## 1. Introduction

The ceramic phases of the general formula *A*_2_*M*_3_O_12_ and the related families, such as *ABM*_3_O_12_, *ABM*_2_*X*O_12_ and *A*_2_*MX*_2_O_12_, have potential for thermal shock resistance applications, since they can present near-zero thermal expansion over wide and technologically relevant temperature intervals, including room temperature (RT) [1]. A potential mechanism for tuning the coefficient of thermal expansion (CTE) in these families is through *A*^3+/4+^ and *B*^2+/3+^ cation variation, which leads to changes of rigidity of the framework octahedra [2,3]. More flexible octahedra enhance negative thermal expansion [3]. 

Based on the knowledge of coefficients of thermal expansion and monoclinic-to-orthorhombic phase transition temperatures in Al_2_W_3_O_12_ and In_2_W_3_O_12_ [4,5,6,7], the Al_2−*x*_In*_x_*W_3_O_12_ system warrants attention. The end member, Al_2_W_3_O_12_, presents a low positive CTE of 1.51 × 10^−6^ K^−1^, as measured by X-ray powder diffraction (XRPD) [5] and 1.17 × 10^−6^ K^−1^ from dilatometry [6], as well as monoclinic-to-orthorhombic phase transition temperatures reported at −6 °C [4] and −22 °C [8]. On the other hand, both positive (3.1 × 10^−6^ K^−1^ from XRPD [9]) and negative (−3.0 × 10^−6^ K^−1^ from dilatometry [10]) CTEs were reported for In_2_W_3_O_12_. The temperature of its monoclinic-to-orthorhombic phase transition was reported to be between 200 °C and 250 °C [4,7,9]. This transition is important because in this family the monoclinic phase exhibits normal positive CTEs, whereas the orthorhombic phase has low or negative CTEs.

Despite the promising properties of the end members, reports on the Al_2−*x*_In*_x_*W_3_O_12_ system are scant [11,12,13,14,15]. Evans et al. presented a brief report [11] of dilatometry measurements, showing near-zero to low-positive CTEs for this system in the range of 0.2 ≤ *x* ≤ 0.5, while Mary and Sleight [12], using the same technique, reported zero thermal expansion for the AlInW_3_O_12_ phase within the temperature range between RT and 727 °C. In a more recent study, Tzvetkov et al. [13] reported a Rietveld XRPD study of Al_1.5_In_0.5_W_3_O_12_ and AlInW_3_O_12_, suggesting both materials were orthorhombic (space group 60, *Pnca*) at RT. In fact, based on visual inspection of XRPD patterns, those authors attributed the orthorhombic structure at RT to all members of the Al_2−*x*_In*_x_*W_3_O_12_ system from *x* = 0.2 to 1.4. In a later study [14], the same group reported co-precipitation synthesis of different phases from the Al_2−*x*_In*_x_*W_3_O_12_ system, although in contrast with their previous report, the authors stated that the phases for *x* ≥ 1.3 were monoclinic at RT. 

To the best of the authors’ knowledge, there are no thorough non-ambient studies on this system. Furthermore, there is a lack of knowledge on CTE, phase transition and hygroscopicity within the Al_2−*x*_In*_x_*W_3_O_12_ system, and there are discrepancies in the literature [13,14] concerning the RT crystal structure of some compositions from this system. In addition, there are no comparative studies of intrinsic (XRPD) and extrinsic (dilatometry) CTE for the Al_2−*x*_In*_x_*W_3_O_12_ system, so it is not clear whether this system could be used to achieve near-zero CTE phases; therefore, the evaluation the of suitability of the Al_2−*x*_In_*x*_W_3_O_12_ (*x* = 0.2–1.0) system for near-zero thermal expansion materials was the main knowledge gap to be filled by the present study.

Accordingly, the Al_2−*x*_In*_x_*W_3_O_12_ system was thoroughly studied using high-temperature XRPD, dilatometry, differential scanning calorimetry and thermogravimetric analysis over the composition range of 0.2 ≤ *x* ≤ 1, and with wide temperature intervals, ranging from cryogenic to high temperatures.

## 2. Experimental 

### 2.1. Synthesis of Al_2−x_In_x_W_3_O_12_ Powders through Modified Reverse-Strike Co-Precipitation 

Al(NO_3_)_3_·*x*H_2_O, In(NO_3_)_3_·*x*H_2_O (Alfa Aesar, purity ≥ 99%) and Na_2_WO_4_·2H_2_O (Sigma Aldrich, St. Louis, MO, USA, purity ≥ 99%) were used as purchased.

Four precursor powders of Al_2−*x*_In*_x_*W_3_O_12_ with the nominal chemical compositions *x* = 0.2, 0.4, 0.7 or 1 were prepared using a modified reverse-strike approach [16] through simultaneous dripping of stochiometric aqueous 0.1 M solutions of Al(NO_3_)_3_·*x*H_2_O and In(NO_3_)_3_·*x*H_2_O into 0.1 M Na_2_WO_4_·2H_2_O with stirring at 600 rpm (Fisam, model 752a) at RT. The pH was not adjusted and naturally tended to a value close to 4 for each mixture (pH = 4.1 for *x* = 0.2, 4.0 for *x* = 0.4, 3.9 for *x* = 0.7, 3.7 for *x* = 1). A white precipitate was instantaneously formed with the addition of nitrates for all chemical compositions. The precipitates were recovered by centrifugation immediately after the formation of precipitates, using an NT 810 centrifuge (Novatecnica, Piracicaba, Brazil) at 4000 rpm, then afterwards were washed three times with anhydrous ethanol (Vetec, 99.9%). 

The wet white precipitates were microwave oven dried for 3 min. The dried white precipitate was heated to 600 °C for 1 h and then calcined at 950 °C for 10 h to obtain powders with larger crystallites, which were well suited for XRPD.

### 2.2. Characterization of Al_2−__x_In_x_W_3_O_12_ Powders

To determine intrinsic CTEs and information concerning the possible phase transition from the monoclinic to orthorhombic form, room- and high-temperature X-ray powder diffraction (HT-XRPD) measurements were carried out using a Bruker D8 Advance diffractometer with Cu_α_ radiation (λ = 1.5418 Å), with a 2θ scanning step size of 0.01° and counting time of 2 s per step. High-temperature experiments were performed using a high-temperature Anton-Paar XRK900 camera. HT-XRPD data were acquired at 50, 100, 150, 200, 250, 300, 350 and 400 °C. Intrinsic CTEs (linear and axial) were calculated using the unit-cell parameters (with the standard uncertainties at the fourth decimal place) determined by Le Bail refinement, using Topas-Academic software. The standard deviations for the intrinsic CTE were calculated from the linear fit in the temperature range from room temperature to 400 °C.

Extrinsic CTEs for Al_2−*x*_In*_x_*W_3_O_12_ samples in the form of bars were determined using a DIL 402C NETZSCH dilatometer. Green bodies (bars) were prepared using 250 MPa pressing of powders obtained by co-precipitation, mixed with 1 mass% polyethylene glycol and 1 mass% polyvinyl alcohol. Afterwards, green bodies were sintered at 950 °C for 10 h in air. High relative densities (>96% of theoretical density) were obtained for the as-sintered bodies, as measured using the Archimedes method (Supporting Information, Table S1). Dilatometric curves were acquired on sintered bars (diameter 6.5 mm; length 8 mm) on heating from RT to 500 °C at a rate of 5 K min^−1^ and on cooling at a rate of 3 K min^−1^. Three measurements were performed for each composition and standard deviations were calculated. The dilatometer had been previously calibrated for CTE measurements using Al_2_O_3_ standard sample supplied by NETZSCH. CTEs were calculated from heating curves from RT to 500 °C.

Thermogravimetric analyses (TGA) were performed on a Perkin–Elmer STA-6000 simultaneous thermal analyzer. Samples (~20 mg) were heated from RT to 950 °C at 10 K min^−1^ in synthetic air at a flow rate of 20 mL min^−1^.

Differential scanning calorimetry (DSC) analyses were conducted in a TA Instruments Q200, equipped with a liquid N_2_ cooling head under He purge gas (25 mL min^−1^), with a heating rate of 20 K min^−1^ over the temperature ranges from −100 °C to 0 °C and from 0 °C to 300 °C. 

## 3. Results and Discussion

**Figure 1:**
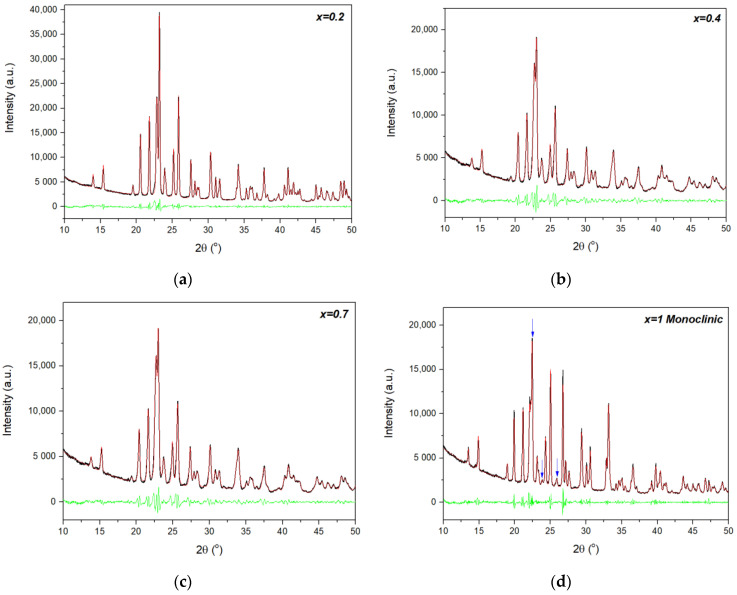
XRPD patterns of Al_2−*x*_In*_x_*W_3_O_12_ powders at RT, illustrating Le Bail fits to the orthorhombic *Pbcn* space group for the compositions (**a**) *x* = 0.2, (**b**) 0.4 and (**c**) 0.7 and to the monoclinic *P2_1_/a* space group for (**d**) *x* = 1 (to highlight the differences between orthorhombic and monoclinic patterns, the 2θ angle range between 10° and 50° is presented). Experimental profiles are represented by black lines, calculated profiles by red lines and difference profiles by green lines. (**d**) The arrows show typical diffraction lines for the monoclinic system in the *A*_2_*M*_3_O_12_ family, which are absent in the orthorhombic system.

XRPD patterns at RT of four different phases from the Al_2−*x*_In*_x_*W_3_O_12_ system (*x* = 0.2, 0.4, 0.7 and 1) are presented in Figure 1. All four powders were single-phased. The unit-cell parameters of Al_2−*x*_In*_x_*W_3_O_12_ phases at RT are presented in the Supporting Information, Table S2. Le Bail refinements of the patterns of the *x* = 0.2, 0.4 and 0.7 materials fit very well within the orthorhombic system (space group 60, *Pbcn*); however, the XRPD pattern of *x* = 1 (Figure 1d) was slightly different from the results for the three phases with a lower In^3+^ content. It was very similar to what would be expected for the orthorhombic *Pbcn* space group, but presented additional features, namely two extra diffraction lines situated at 23.8° and 25.9° (2θ), while the most intense peak, close to 22.4° (2θ), was not divided into two separate diffraction lines as it was for orthorhombic system (Figure 1a–c). These features have been attributed in the literature to the monoclinic system (space group 12, *P2_1_/a*) [17,18] or *P2_1_/n* (space group 14) [8,19]; therefore, the XRPD pattern for *x* = 1 was adjusted to the monoclinic system, which described all experimental diffraction lines very well. As such, the XRPD patterns at RT (Figure 1) suggested that AlInW_3_O_12_ is monoclinic at RT and not orthorhombic, as previously proposed [13,14].

**Figure 2:**
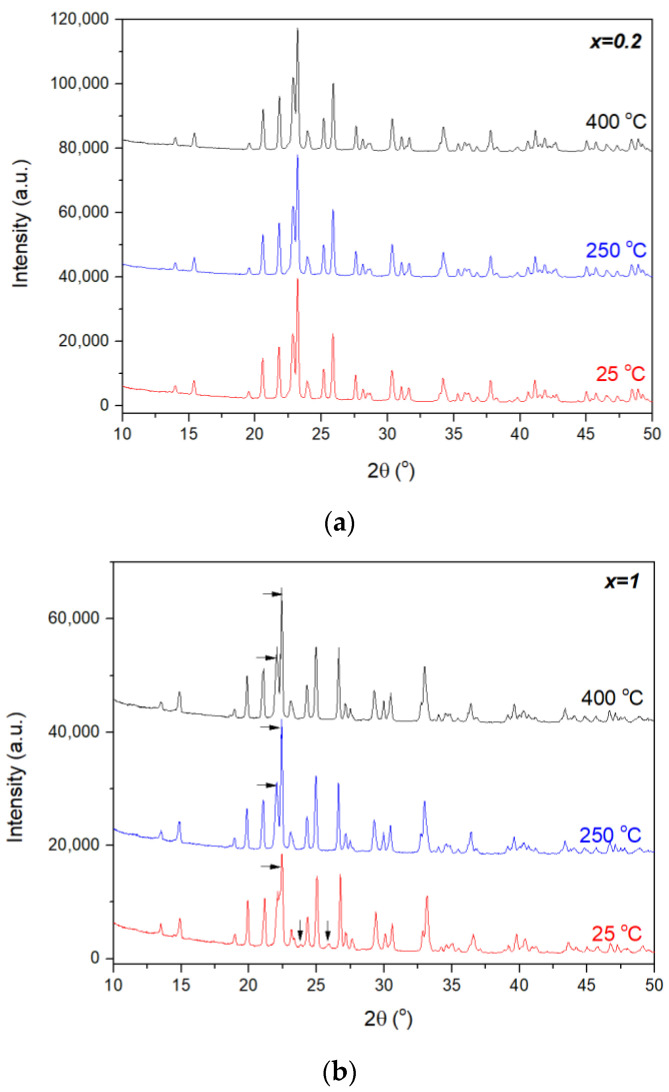
HT-XRPD patterns of Al_2−*x*_In*_x_*W_3_O_12_ materials for (**a**) *x* = 0.2 (orthorhombic) and (**b**) *x* = 1 (illustrating the phase transition from a monoclinic to orthorhombic structure at 250 °C). (**b**) The arrows at RT show typical features for the monoclinic system in the *A*_2_*M*_3_O_12_ family, while at higher temperatures the arrows mark separation of the most intense peak into two diffraction lines, which is a feature of an orthorhombic pattern for the *A*_2_*M*_3_O_12_ family.

HT-XRPD patterns of *x* = 0.2 and *x* = 1 phases are depicted in more detail in Figure 2, while complete sets of diffraction patterns up to 400 °C for all phases are available in Supporting Information, Figure S1. Note that the XRPD pattern of the *x* = 1 phase changed to orthorhombic at 250 °C, as evidenced by the disappearance of the two diffraction lines at 23.8° and 25.9° (2θ), as well as the most intense peak, close to 22.4° (2θ), separating into two adjacent diffraction lines. 

**Figure 3:**
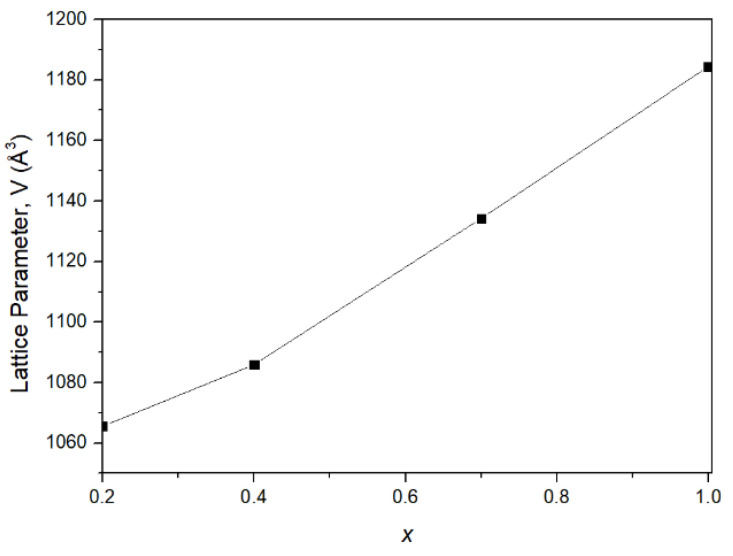
Orthorhombic unit-cell volumes at 250 °C as a function of In^3+^ content (*x*) in Al_2−*x*_In*_x_*W_3_O_12_. Standard deviations are smaller than symbol size.

Proof of In^3+^ incorporation into the orthorhombic crystal structure, in accordance with the nominal chemical compositions of Al_2−*x*_In*_x_*W_3_O_12_ (*x* = 0.2, 0.4, 0.7 and 1), is evident from the isothermal plot constructed for 250 °C, which obeys Vegard’s law [20] (Figure 3). A near linear unit-cell volume increase with the increase of In^3+^ content (*x*) in the orthorhombic Al_2−*x*_In*_x_*W_3_O_12_ phase correlates well with the larger radius of In^3+^ (0.80 Å) in comparison to Al^3+^ (0.535 Å) [21].

Table 1 presents linear and axial CTEs for all four Al_2−*x*_In*_x_*W_3_O_12_ compositions over the temperature interval between RT and 400 or 500 °C. The plots of natural logarithms of unit-cell parameters (*a*, *b* and *c*) and unit-cell volumes as functions of temperature are available in the Supporting Information, Figure S2. Axial CTEs along the *b*- and *c*-axes increased monotonically with increasing *x* up to *x* =0.7, while the axial CTE along the *a*-axis varied up to *x* = 0.7 in a non-monotonical manner. This discrepancy should be verified by higher-resolution synchrotron radiation. Linear CTEs of the *x* = 0.2, 0.4 and 0.7 phases are positive and increase with increasing In^3+^ content (*x*), appearing more in line with the more positive intrinsic CTE of the end-member In_2_W_3_O_12_ (3.1 × 10^−6^ K^−1^), as reported by Baiz et al. [9], than the reported negative linear CTE of In_2_W_3_O_12_ [10]; therefore, the phases in the range between *x* = 0.2 and 0.7 showed a tendency of increased linear CTE in comparison to pure Al_2_W_3_O_12_. Judging from this behavior, it would not be possible to obtain near-zero CTE at RT and higher within the Al_2−*x*_In_*x*_W_3_O_12_ system. The axial CTEs were more positive along the *a*- and *b*-axes and negative or low positive along the *c*-axes. The *x* = 1 phase showed a high linear CTE in the monoclinic form (~10^−5^ K^−1^) from RT to 100 °C, in agreement with the literature [22,23], while above 250 °C when orthorhombic it presented a near-zero CTE (−7.9 × 10^−7^ K^−1^).

**Figure 4:**
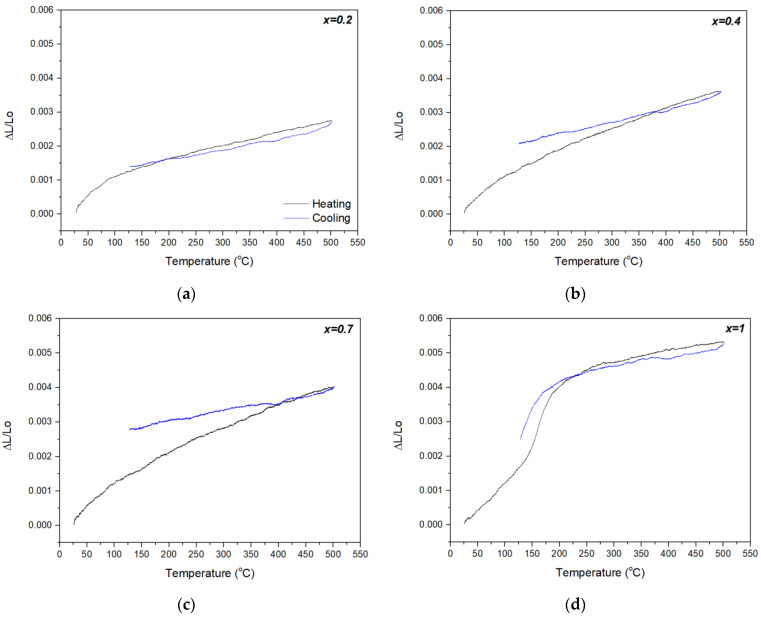
Dilatometric curves of the materials in the family of Al_2−*x*_In_*x*_W_3_O_12_: (**a**) *x* = 0.2; (**b**) *x* = 0.4; (**c**) *x* = 0.7; (**d**) *x* = 1.

Dilatometric curves for the *x* = 0.2, 0.4 and 0.7 materials (Figure 4a–c) showed positive linear extrinsic CTEs, higher than those measured by XRPD (Table 1). These orthorhombic phases demonstrated the tendency for increased CTE with the increase of In^3+^ content (*x*), as also observed for the intrinsic linear CTEs. The increased extrinsic CTEs relative to the intrinsic CTEs might be partially understood as a consequence of the anisotropy of elastic constants in the *A*_2_*M*_3_O_12_ family [24]. In addition, dilatometry clearly confirmed the phase transition in *x* = 1 between 150 and 220 °C (Figure 4d), from the denser monoclinic polymorph to the more open (higher volume) orthorhombic form [25]. 

**Figure 5:**
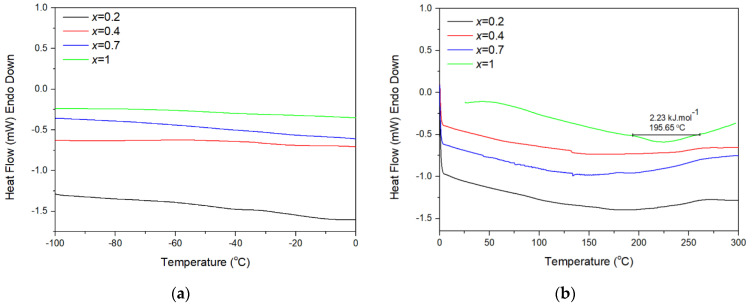
DSC curves for the *x* = 0.2, 0.4, 0.7 and 1 compositions in the family of Al_2−*x*_In_*x*_W_3_O_12_ at different temperatures: (**a**) cryogenic temperatures: (**b**) ambient and high temperatures. For *x* = 1, the onset temperature of the monoclinic-to-orthorhombic phase transition and the enthalpy of phase transition are presented as well.

DSC curves at cryogenic, ambient and above ambient temperatures are presented in Figure 5. Figure 5b shows an endothermic peak (with the onset temperature of ~195 °C) as another indication of the monoclinic-to-orthorhombic phase transition in the *x* = 1 material. The enthalpy change at the phase transition (2.23 kJ mol^−1^) is in accordance with the low enthalpy changes usually measured for displacive monoclinic-to-orthorhombic phase transitions in this ceramic family [26], owing to small differences in internal energies and molar volumes between the two phases. The other three DSC curves are practically featureless in the vast temperature range from −100 °C to 300 °C. Two faint kinks registered at ~130 °C in the DSC curves of *x* = 0.4 and 0.7 materials were likely artefacts, as they were not corroborated either by HT-XRPD or dilatometric measurements. 

TGA curves (see Supporting Information, Figure S3) of all four phases revealed mass losses <1 mass% over the temperature range from RT to 950 °C, classifying these materials as non-hygroscopic. 

The analyses of data acquired by XRPD, dilatometry and DSC strongly support the finding of the monoclinic structure (*P2_1_/a* space group) at RT for the *x* = 1 material, which transforms to being orthorhombic at ca. 200 °C. These findings are not in line with the observations from earlier reports [12,13,14]. The inconsistencies can mostly likely be attributed to erroneous interpretation and analysis of XRPD patterns at RT [13,14]. A similar inconsistency was reported by Truitt et al. [27] for Fe_1.5_Y_0.5_Mo_3_O_12_, for which the orthorhombic system had been previously assigned down to −170 °C [28]. 

In the present study, no phase transition to a monoclinic (or other) phase was identified for *x* = 0.2, *x* = 0.4 or *x* = 0.7 over the temperature range of −100 °C to 300 °C (Figure 4 and Figure 5). This feature of Al_2−*x*_In*_x_*W_3_O_12_ does not follow the electronegativity rule [4], contrary to the majority of *A*_2_*M*_3_O_12_ materials [1,29]. By this rule, phase transition temperatures for the intermediate phases in the Al_2−*x*_In*_x_*W_3_O_12_ system would have values between the phase transition temperatures of the end members, i.e., between −22 °C and 250 °C. Other notable exemptions from the electronegativity rule are AlScMo_3_O_12_ [27] and the Al_2−*x*_Sc*_x_*W_3_O_12_ system [30,31]. Truitt et al. [27] proposed that a large difference in radii of the cations occupying octahedral position in AlScMo_3_O_12_ (Al/Sc ratio = 0.72) could contribute to such a finding. This difference is even higher for Al/In (ratio 0.675) and might explain the suppression of the transition to the monoclinic form in *x* = 0.2, *x* = 0.4 and *x* = 0.7 materials, but is not in the line with the observed phase transition for AlInW_3_O_12_ (at ~200 °C), which does fit the electronegativity rule. The unusual suppression of the orthorhombic-to-monoclinic transition in the present results for Al_2−*x*_In*_x_*W_3_O_12_ (*x* = 0.2, 0.4 and 0.7) and in a few other systems, namely AlScMo_3_O_12_ [27] and Al_2−*x*_Sc*_x_*W_3_O_12_ [30,31]_,_ still does not have a satisfactory explanation. 

Intrinsic CTEs of the *x* = 0.2 and *x* = 0.4 phases are between the values of the end members, while the linear CTE for *x* = 0.7 is higher than for the In_2_W_3_O_12_ end member [9]. This behavior of CTEs has been observed in related materials [29,32]. It is also relevant to note that the linear CTE of In_2_W_3_O_12_ is positive [9] and very different from the negative linear CTE reported for its molybdate counterpart, In_2_Mo_3_O_12_ [23]. Linear CTEs of *A*_2_*M*_3_O_12_ phases were initially correlated with the cationic radii of *A^3+^*, but now the octahedra volume distortion model [2,3] is considered to be more accurate and grounded in explaining the behavior of the linear CTE. The linear CTE of In_2_W_3_O_12_ does not fit with cationic radii rationalization and is yet to be assessed in terms of the octahedra volume distortion model; however, it is confirmed by our results (Table 1 and Supporting Information, Table S1) that the *x* = 0.2, 0.4 and 0.7 materials in the Al_−*-x*_In_*x*_W_3_O_12_ family have low positive thermal expansion, as is the case for In_2_W_3_O_12_. 

On the other hand, orthorhombic AlInW_3_O_12_ showed a near-zero thermal expansion above 250 °C, in contrast with the other three compositions (*x* = 0.2, 0.4 and 0.7). This peculiar behavior of AlInW_3_O_12_ was also noted in the phase transition, since *x* = 1 was the only composition exhibiting a phase transition to the monoclinic form in accordance with the electronegativity rule. 

## 4. Conclusions

This study showed that the Al_2−*x*_In*_x_*W_3_O_12_ system is not promising for designing materials with near-zero thermal expansion over a wide range that includes RT. 

In contrast with some previous reports, this study provided solid proof that AlInW_3_O_12_ is monoclinic at RT and transforms to the orthorhombic form at ca. 200 °C. In addition, there was no evidence of orthorhombic-to-monoclinic phase transitions for *x* = 0.2, *x* = 0.4 and *x* = 0.7 materials in the Al_2−*x*_In_*x*_W_3_O_12_ family in the temperature interval from −100 °C to 500 °C.

Linear intrinsic CTEs of *x* = 0.2, *x* = 0.4 and *x* = 0.7 are low and positive between RT and 400 °C (ranging from 1.88 × 10^−6^ to 3.88 × 10^−6^ K^−1^), and higher than the linear intrinsic CTE of the Al_2_W_3_O_12_ end member in the Al_2−*x*_In_*x*_W_3_O_12_ series.

The suppression of the orthorhombic-to-monoclinic phase transition in *x* = 0.2, *x* = 0.4 and *x* = 0.7 materials does not align with expectations based on the electronegativity rule. This finding, along with other literature reports, indicates that there are likely other mechanisms governing the orthorhombic-to-monoclinic phase transition in addition to the electronegativity of *A^3+^* in octahedral sites.

## Figures and Tables

**Table 1:** Intrinsic and extrinsic CTEs by axis (*α_a_*, *α_b_*, *α_c_*) and average linear CTE (*α****_l_***) in the Al_2−*x*_In_*x*_W_3_O_12_ system (*Pbcn* space group for the orthorhombic phase and *P2_1_/a* for the monoclinic phase) with their standard deviations (standard deviations for *x* = 1 in monoclinic form are one order of magnitude higher than for others, since only three points were considered). Standard deviations for CTEs of the end-members are not reported in the literature [5,9]. Results not referenced to the literature are from the present study. RT refers to room temperature.

*x* (In^3+^ Content in Chemical Formula)	*α_a_* [K^−1^]	*α_b_* [K^−1^]	*α_c_* [K^−1^]	*α_l_* [K^−1^]	Temperature Range [°C]	Ref.
0	5.94 × 10^−6^	−0.994 × 10^−6^	−1.31 × 10^−6^	1.51 × 10^−6^	RT–800 (XRPD)	[5]
0.2	4.98 × 10^−6^ ±1.3 × 10^−7^	9.47 × 10^−7^ ±1.3 × 10^−7^	−2.93 × 10^−7^ ±1.5 × 10^−7^	1.88 × 10^−6^ ±1.4 × 10^−7^ 4.96 × 10^−6^ ±1.8 × 10^−7^	RT–400 (XRPD) RT–500 (dilatometry)	-
0.4	3.80 × 10^−6^ ±2.2 × 10^−7^	2.09 × 10^−6^ ±2.4 × 10^−7^	8.77 × 10^−7^ ±2.7 × 10^−7^	2.25 × 10^−6^ ±2.4 × 10^−7^ 6.29 × 10^−6^ ±7.0 × 10^−7^	RT–400 (XRPD) RT–500 (dilatometry)	-
0.7	5.52 × 10^−6^ ±1.2 × 10^−7^	3.92 × 10^−6^ ±3.2 × 10^−7^	2.22 × 10^−6^ ±2.3 × 10^−7^	3.88 × 10^−6^ ±2.2 × 10^−7^ 6.00 × 10^−6^ ±7.0 × 10^−7^	RT–400 (XRPD) RT–500 (dilatometry)	-
1 (*P2_1_/a*))	1.83 × 10^−5^ ±7.5 × 10^−6^	1.47 × 10^−5^ ±4.5 × 10^−6^	2.63 × 10^−5^ ±8.1 × 10^−6^	1.88 × 10^−5^ ±7.7 × 10^−6^	RT–100 (XRPD)	-
1 (*Pbcn*)	8.47 × 10^−7^ ±2.9 × 10^−7^	−9.32 × 10^−7^ ±4.0 × 10^−7^	−2.27 × 10^−6^ ±4.7 × 10^−7^	−7.87 × 10^−7^ ±3.9 × 10^−7^ 3.18 × 10^−6^ ±1.6 × 10^−7^	250–400 (XRPD) 250–500 (dilatometry)	-
2 (*Pbcn*)	−3.1 × 10^−6^	11 × 10^−6^	1.6 × 10^−6^	3.1 × 10^−6^	250–600 (XRPD)	[9]

## Data Availability

The data underlying this article will be shared on reasonable request from the corresponding author.

## Supplementary Materials

The following are available online at https://www.mdpi.com/article/10.3390/ma14144021/s1, Figure S1: HT-XRPD patterns of (a) *x* = 0.2; (b) *x* = 0.4; (c) *x* = 0.7 and (d) *x* = 1 phase, from RT to 400 °C, Figure S2-1: Natural logarithmic variations of unit-cell parameters (a, b and c) and unit-cell volume vs. temperature for *x* = 0.2 phase. (d), Figure S2-2: Natural logarithmic variations of unit-cell parameters (a, b and c) and unit-cell volume vs. temperature for *x* = 0.4 phase (d). Figure S2-3: Natural logarithmic variations of unit-cell parameters (a, b and c) and unit-cell volume vs. temperature for *x* = 0.7 phase (d), Figure S2-4: Natural logarithmic variations of unit-cell parameters (a, b and c) and unit-cell volume vs. temperature for *x* = 1 phase (monoclinic form) (d), Figure S2-5: Natural logarithmic variations of unit-cell parameters (a, b and c) and unit-cell volume vs. temperature for *x* = 1 phase (orthorhombic form) (d), Figure S3: TGA curves of phases in Al_2−*x*_In_*x*_W_3_O_12_ system: (a) *x* = 0.2; (b) *x* = 0.4; (c) *x* = 0.7 and (d) *x* = 1. Table S1: Theoretical, real and relative densities of Al_2−*x*_In_*x*_W_3_O_12_ phases with the nominal chemical compositions *x* = 0.2; *x* = 0.4; *x* = 0.7 and *x* = 1, Table S2: Unit-cell parameters at RT in Al_2−*x*_In_*x*_W_3_O_12_ system.

## References

[1] Liu H., Sun W., Zhang Z., Lovings L., Lind C. (2021). Thermal Expansion Behavior in the A_2_M_3_O_12_ Family of Materials. Solids.

[2] Marinkovic B.A., Ari M., de Avillez R., Rizzo F., Ferreira F.F., Miller K.J., Johnson M.B., White M.A. (2009). Correlation between AO_6_ Polyhedral Distortion and Negative Thermal Expansion in Orthorhombic Y_2_Mo_3_O_12_ and Related Materials. Chem. Mater..

[3] Romao C.P., Perras F.A., Werner-Zwanziger U., Lussier J.A., Miller K.J., Calahoo C.M., Zwanziger J.W., Bieringer M., Marinkovic B.A., Bryce D.L. (2015). Zero Thermal Expansion in ZrMgMo_3_O_12_: NMR Crystallography Reveals Origins of Thermoelastic Properties. Chem. Mater..

[4] Sleight A., Brixner L. (1973). A new ferroelastic transition in some A_2_(MO_4_)_3_ molybdates and tungstates. J. Solid State Chem..

[5] Woodcock D.A., Lightfoot P., Ritterb C. (2000). Negative Thermal Expansion in Y_2_(WO_4_)_3_. J. Solid State Chem..

[6] Imanaka N., Hiraiwa M., Adachi G., Dabkowska H., Dabkowski A. (2000). Thermal contraction behavior in Al_2_(WO_4_)_3_ single crystal. J. Cryst. Growth.

[7] Sivasubramanian V., Ravindran T.R., Nithya R., Arora A.K. (2004). Structural phase transition in indium tungstate. J. Appl. Phys..

[8] Hashimoto T., Sugimoto T., Omoto K., Kineri N., Ogata Y., Tsuda K. (2008). Analysis of phase transition and expansion behaviour of Al_2_(WO_4_)_3_ by temperature-regulated X-ray diffraction. Phys. Status Solidi B.

[9] Baiz T.I., Heinrich C.P., Banek N.A., Vivekens B.L., Lind C. (2012). In-situ non-ambient X-ray diffraction studies of indium tungstate. J. Solid State Chem..

[10] Liu H., Zhang Z., Ma J., Jun Z., Zeng X. (2015). Effect of isovalent substitution on phase transition and negative thermal expansion of In_2−x_Sc_x_W_3_O_12_ ceramics. Ceram. Int..

[11] Evans J., Mary T.A. (1997). Sleight, Negative thermal expansion in a large molybdate and tungstate family. J. Solid State Chem..

[12] Mary T.A., Sleight A.W. (1999). Bulk thermal expansion for tungstate and molybdates of the type A_2_M_3_O_12_. J. Mater. Res..

[13] Tzvetkov P., Ivanova D., Kovacheva D., Nikolov V. (2009). Synthesis and powder XRD characterization of Al_2−x_In_x_(WO_4_)_3_ solid solutions. J. Alloy. Compd..

[14] Zhecheva E., Stoyanova R., Ivanova S., Nikolov V. (2010). On the preparation of nanosized Al_2_(WO_4_)_3_ by a precipitation method. Solid State Sci..

[15] Yordanova A.S., Nikolov V.S., Koseva I.I. (2015). Fabrication of high density ceramic from Al_2−X_In_X_(WO_4_)_3_ solid solutions. J. Chem. Technol. Metall..

[16] Pontón P.I., Prisco L.P., Dosen A., Faro G.S., de Abreu M.A., Marinkovic B.A. (2017). Co-precipitation synthesis of Y_2_W_3_O_12_ submicronic powder. Ceram. Int..

[17] Miller K.J., Johnson M.B., White M.A., Marinkovic B.A. (2012). Low-temperature investigations of the open-framework material HfMgMo_3_O_12_. Solid State Commun..

[18] Costa I.L.M., Blair V.L., Paraguassu W., Marinkovic B.A. (2019). Evaluating Al_2−x_GaxW_3_O_12_ system for thermal shock resistance. J. Solid State Chem..

[19] Sugimoto Y., Aoki T., Niwa Y., Hashimoto E., Morito T. (2007). Thermal Expansion and Phase Transition Behavior of Al_2−x_M_x_(WO_4_)_3_ (M = Y, Ga and Sc) Ceramics. J. Ceram. Soc. Jap..

[20] Jacob K.T., Raj S., Rannesh L. (2007). Vegard’s law: A fundamental relation or an approximation?. Int. J. Mater. Res..

[21] Shannon R.D. (1976). Revised effective ionic radii and systematic studies of interatomic distances in halides and chalcogenides. J. Acta Crystallogr. A.

[22] Evans J., Mary T. (2000). Structural phase transitions and negative thermal expansion in Sc_2_(MoO_4_)_3_. Int. J. Inorg. Mater..

[23] Marinkovic B.A., Ari M., Jardim P., de Avillez R.R., Rizzo F., Ferreira F.F. (2010). In_2_Mo_3_O_12_: A low negative thermal expansion compound. Thermochim. Acta.

[24] Romao C.P., Donegan S.P., Zwanziger J.W., White M.A. (2016). Relationships between elastic anisotropy and thermal expansion in A_2_Mo_3_O_12_ materials. Phys. Chem. Chem. Phys..

[25] Prisco L.P., Pontón P., Paraguassu W., Romao C., White M.A., Marinkovic B.A. (2016). Near-zero thermal expansion and phase transition in In_0.5_(ZrMg)_0.75_Mo_3_O_12_. J. Mater. Res..

[26] Varga T., Moats J.L., Ushakov S., Navrotsky A. (2007). Thermochemistry of A_2_M_3_O_12_ negative thermal expansion materials. J. Mater. Res..

[27] Truitt R., Hermes I., Main A., Sendecki A., Lind C. (2015). Low Temperature Synthesis and Characterization of AlScMo_3_O_12_. Materials.

[28] Li Z.Y., Song W.B., Liang E.J. (2011). Structures, Phase Transition, and Crystal Water of Fe_2–x_Y_x_Mo_3_O_12_. J. Phys. Chem. C.

[29] Ari M., Jardim P., Marinkovic B., Rizzo F., Ferreira F.F. (2008). Thermal expansion of Cr_2x_Fe_2−2x_Mo_3_O_12_, Al_2x_Fe_2−2x_Mo_3_O_12_ and Al_2x_Cr_2−2x_Mo_3_O_12_ solid solutions. J. Solid State Chem..

[30] Zhu J., Yang J., Cheng X. (2012). Synthesis and tunable thermal expansion property of Al_2−__δ_Sc_δ_W_3_O_12_. Solid State Sci..

[31] Dasgupta N., Sorge E., Butler B., Wen T.-C., Shetty D.K., Cambrea L.R., Harris D.C. (2012). Synthesis and characterization of Al_2−x_Sc_x_(WO_4_)_3_ ceramics for low-expansion infrared-transmitting windows. J. Mater. Sci..

[32] Liu J., Maynard-Casely H.E., Brand H.E.A., Sharma N. (2021). Sc_1.5_Al_0.5_W_3_O_12_ Exhibits Zero Thermal Expansion between 4 and 1400 K. Chem. Mater..

