# Supplementary material for: Phase Transition and Coefficients of Thermal Expansion in Al2−xInxW3O12 (0.2 ≤ x ≤ 1)
Source: Materials (Basel). 2021 Jul 18;14(14):4021. doi: 10.3390/ma14144021 (PMC8305276; doi:10.3390/ma14144021)
Supplement: Supplementary file 1 [file materials-14-04021-s001.zip › materials-1299126-supplementary.pdf]

Supplementary materials

Phase Transition and Coefficients of Thermal Expansion in $Al_{2-x}In_xW_3O_{12}$ ($0.2 \leq x \leq 1$)

Andrés Esteban Cerón Cortés [1], Anja Dosen [1], Victoria L. Blair [2], Michel B. Johnson [3], Mary Anne White [3,4] and Bojan A. Marinkovic [1,*]

[1] Department of Chemical and Materials Engineering, Pontifical Catholic University of Rio de Janeiro (PUC-Rio), Rio de Janeiro 22453-900, RJ, Brazil; ceron@aluno.puc-rio.br (A.E.C.C.); adosen@puc-rio.br (A.V.D.)
[2] DEVCOM Army Research Laboratory, 6300 Rodman Rd. APG, MD 21005, USA; victoria.l.blair3.civ@mail.mil
[3] Clean Technologies Research Institute, Dalhousie University, Halifax, NS B3H 4R2, Canada; michel.johnson@Dal.Ca (M.B.J.); mary.anne.white@dal.ca (M.A.W.)
[4] Department of Chemistry, Dalhousie University, Halifax, Nova Scotia B3H 4R2, Canada
* Correspondence: bojan@puc-rio.br; Tel.: +55-21-3527-1954

Table S1. Theoretical, real and relative densities of $Al_{2-x}In_xW_3O_{12}$ phases with the nominal chemical compositions x=0.2; x=0.4; x=0.7 and x=1.

x (In^{3+}Content)	Theoretical density (g.cm^{-3})	Density of sintered bodies (g.cm^{-3})	Relative density (%)
0.2	5.085	4.891	96.16
0.4	5.102	5.086	99.68
0.7	5.044	4.939	97.92
1	5.017	4.891	97.50

Table S2. Unit-cell parameters at RT in $Al_{2-x}In_xW_3O_{12}$ system.

Lattice parameter / *x* (In^{3+} Content)	a (Å)	b (Å)	c (Å)	Volume (Å^3)
0.2 (*Pbcn*)	12.69	9.11	9.20	1064.29
0.4 (*Pbcn*)	12.78	9.16	9.26	1083.81
0.7 (*Pbcn*)	12.96	9.29	9.39	1130.62
1 (*P2$_1$/a*)*	16.13	9.49	18.76	2343.85

*β = 125.34°

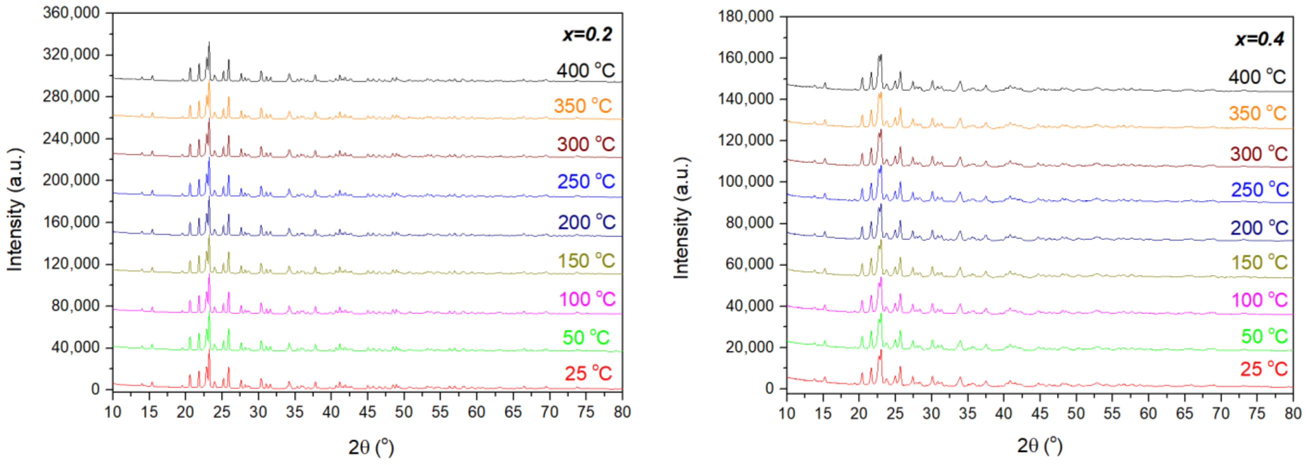

(**a**) (**b**)

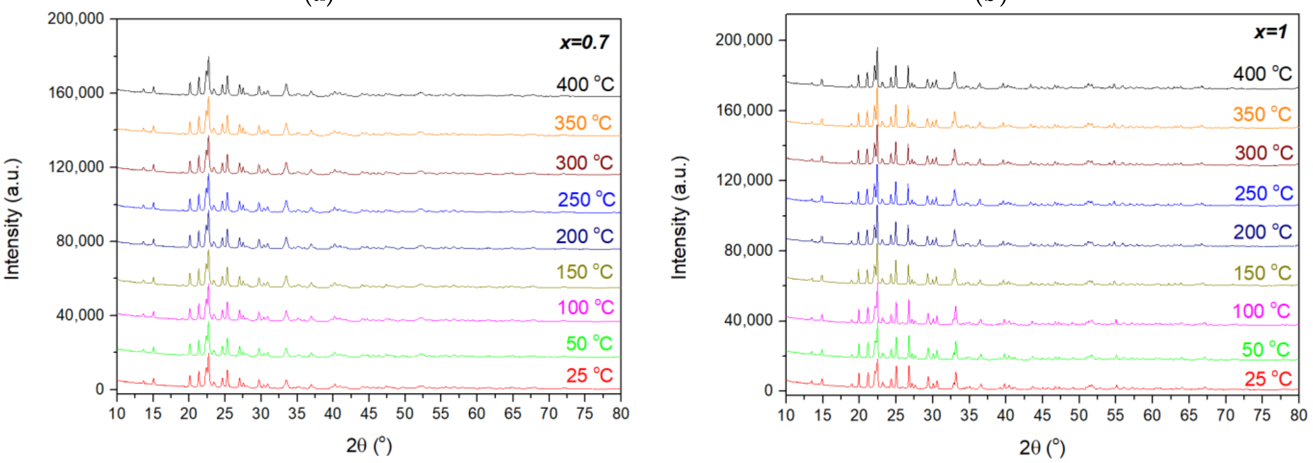

(**c**) (**d**)

Figure S1. HT-XRPD patterns of (**a**) x=0.2; (**b**) x=0.4; (c) x=0.7 and (**d**) x=1 phases, from RT to 400 °C.

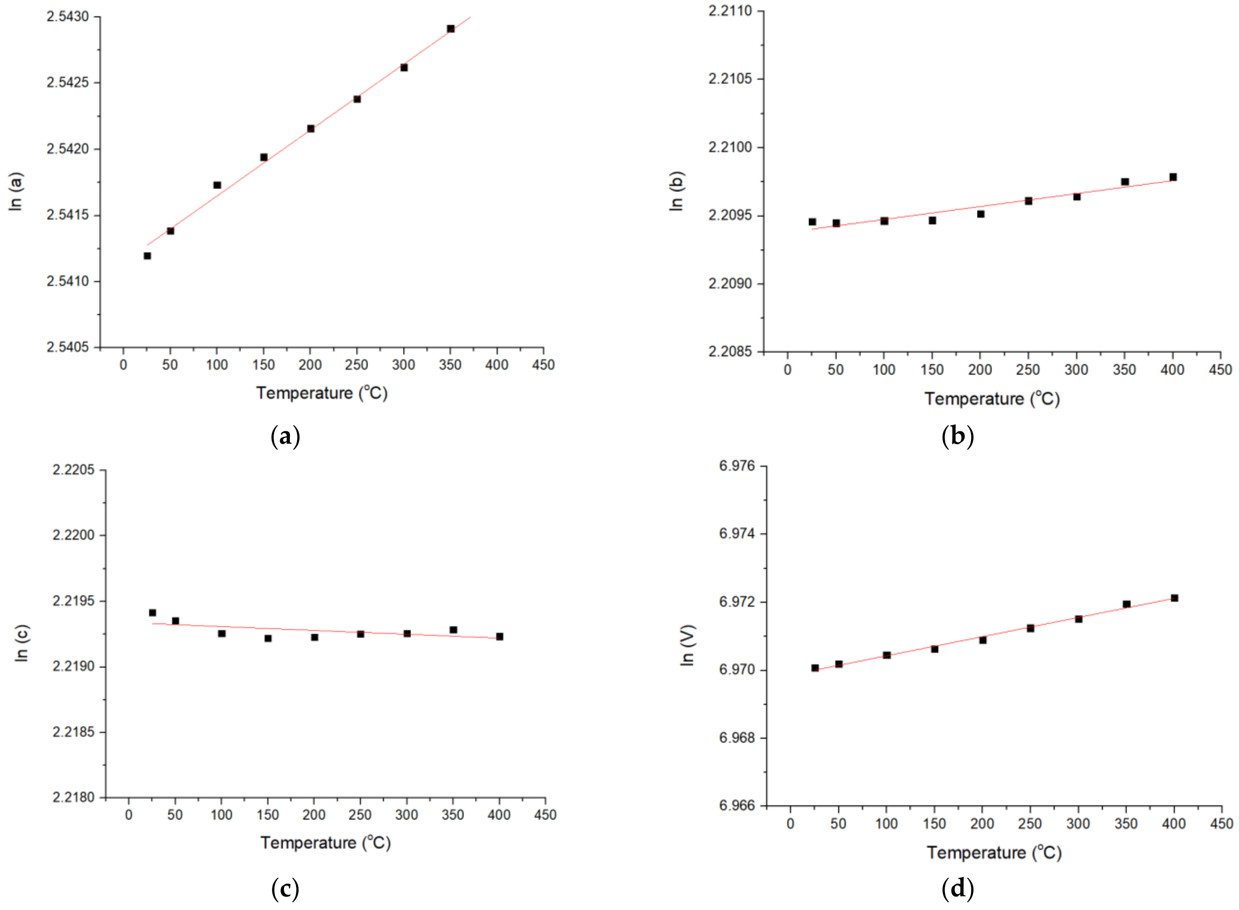

Figure S2-1. Natural logarithmic variations of unit-cell parameters (**a**, **b** and **c**) and unit-cell volume *vs.* temperature for x=0.2 phase (**d**).

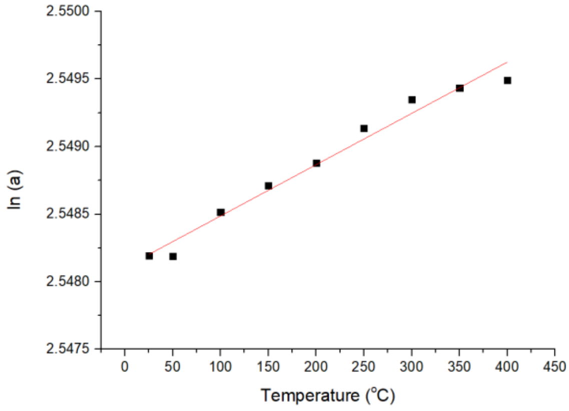

(**a**)

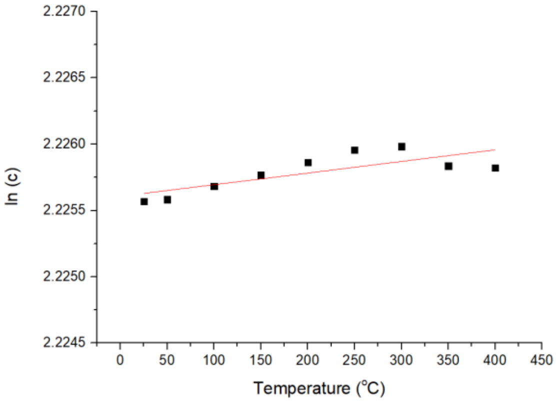

(**b**)

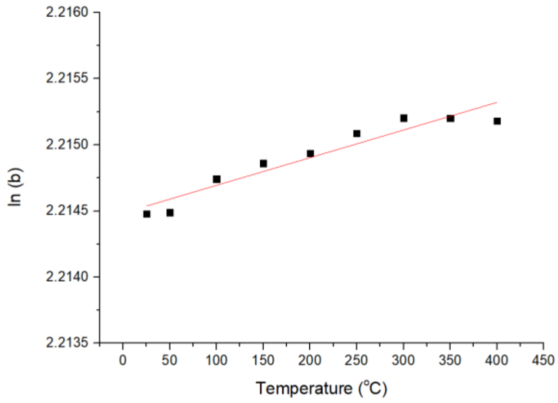

(**c**)

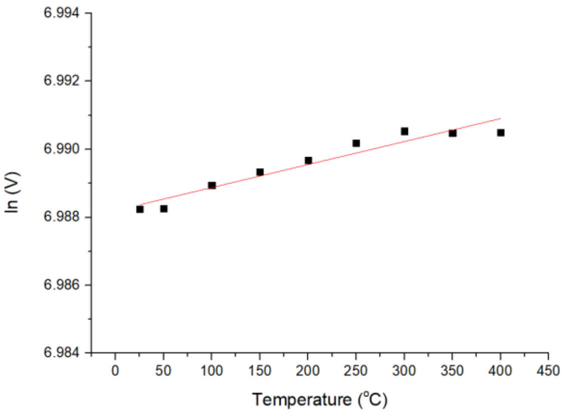

(**d**)

Figure S2-2. Natural logarithmic variations of unit-cell parameters (**a**, **b** and **c**) and unit-cell volume *vs*. temperature for x=0.4 phase (**d**).

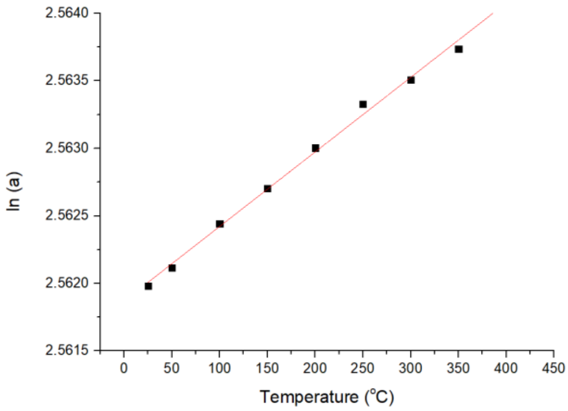

(**a**)

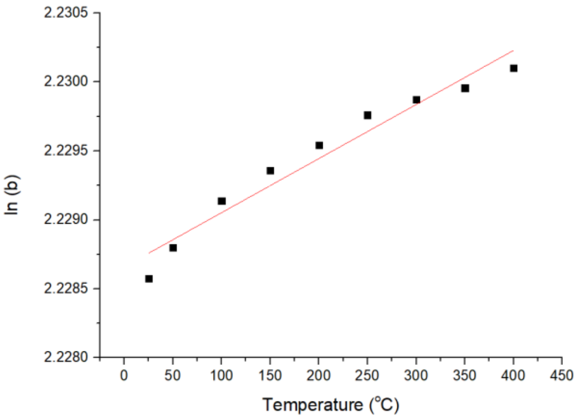

(**b**)

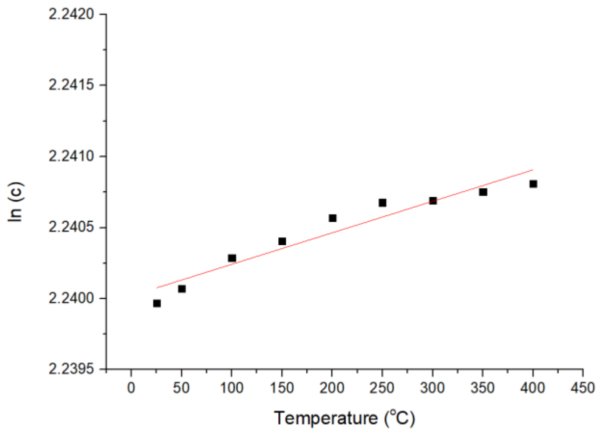

(**c**)

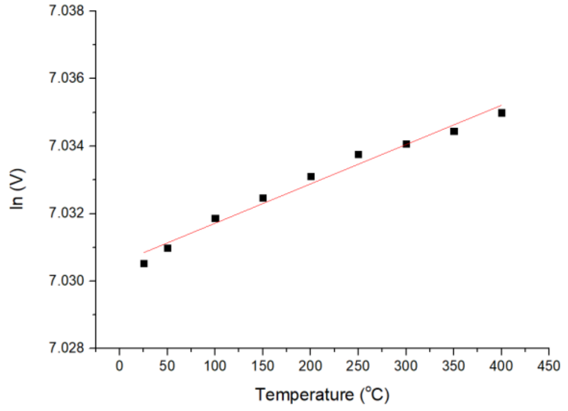

(**d**)

Figure S2-3. Natural logarithmic variations of unit-cell parameters (**a**, **b** and **c**) and unit-cell volume *vs.* temperature for x=0.7 phase (**d**).

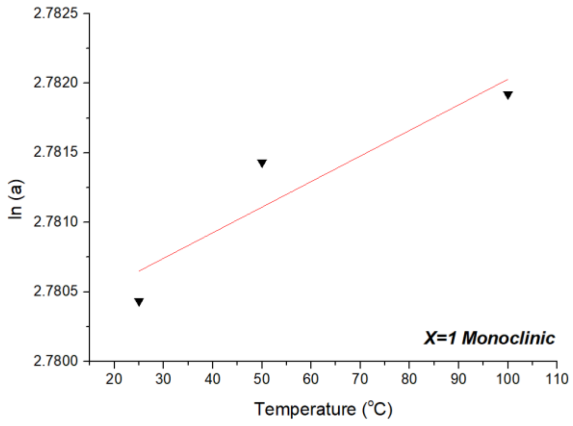

(**a**)

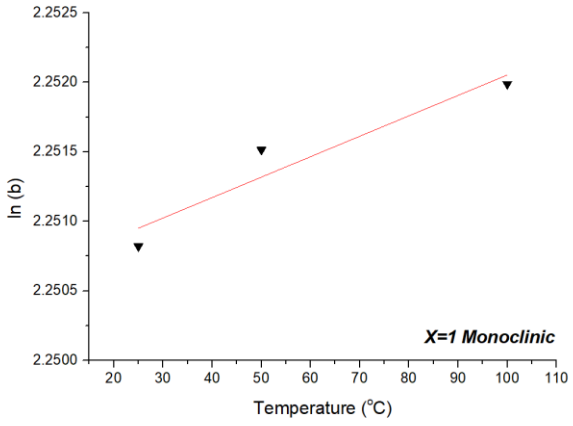

(**b**)

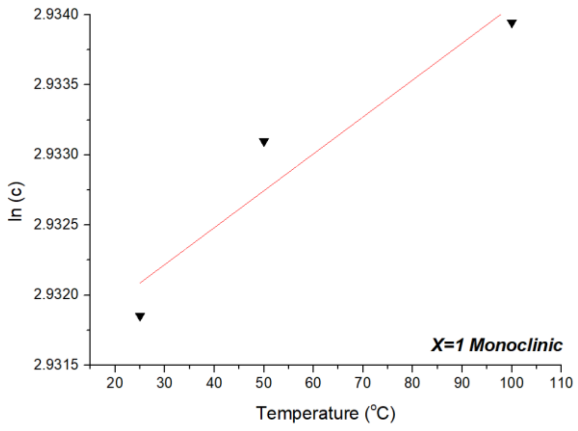

(**c**)

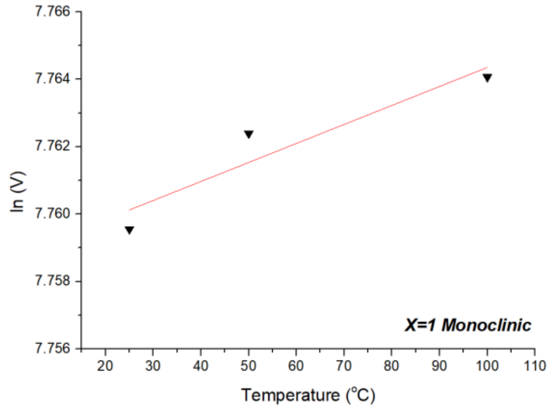

(**d**)

Figure S2-4. Natural logarithmic variations of unit-cell parameters (**a**, **b** and **c**) and unit-cell volume *vs.* temperature for x=1 phase (monoclinic form) (**d**).

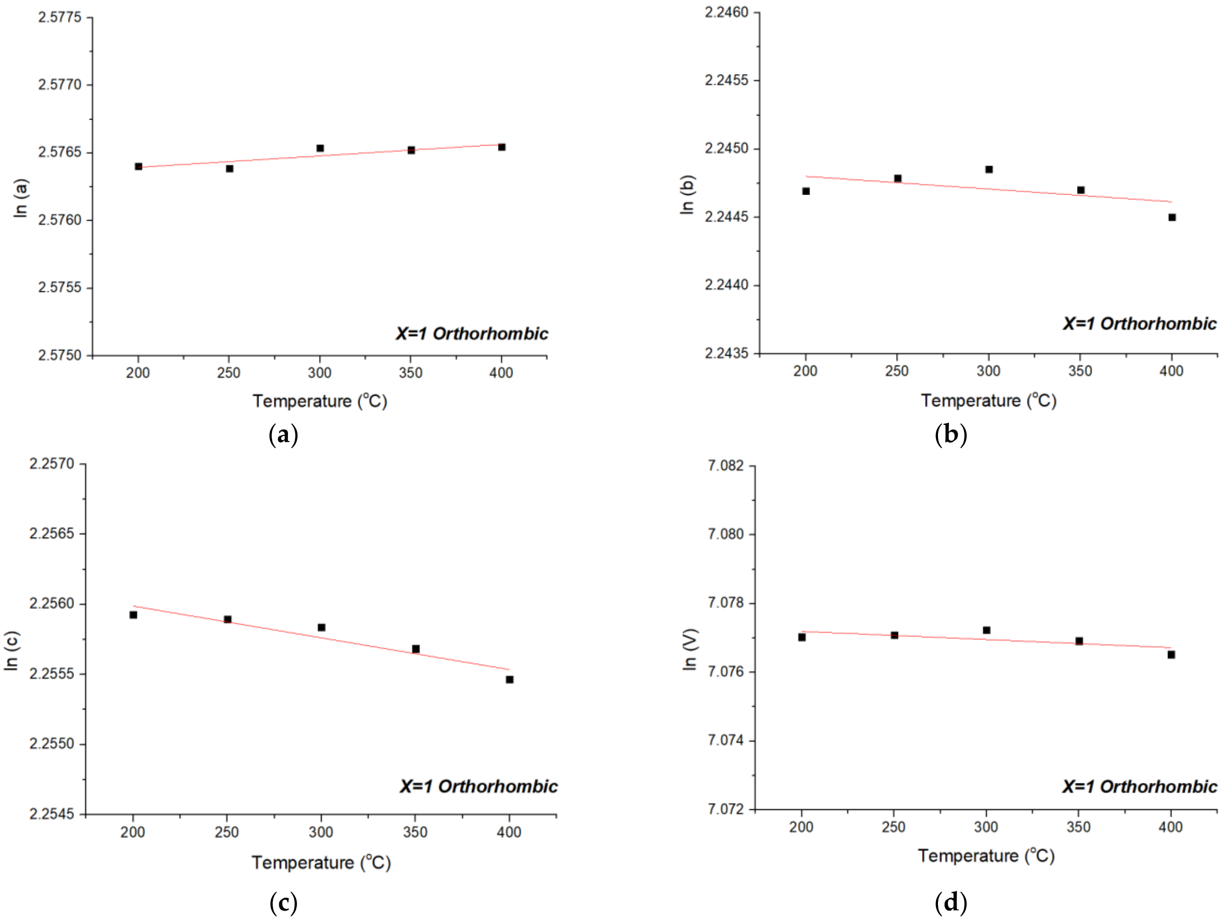

Figure S2-5 Natural logarithmic variations of unit-cell parameters (**a**, **b** and **c**) and unit-cell volume *vs.* temperature for x=1 phase (orthorhombic form) (**d**).

TGA curves show weight losses lower than 1 wt.%, over the temperature range from RT to 950 °C, for all four phases in $Al_{2-x}In_xW_3O_{12}$ system.

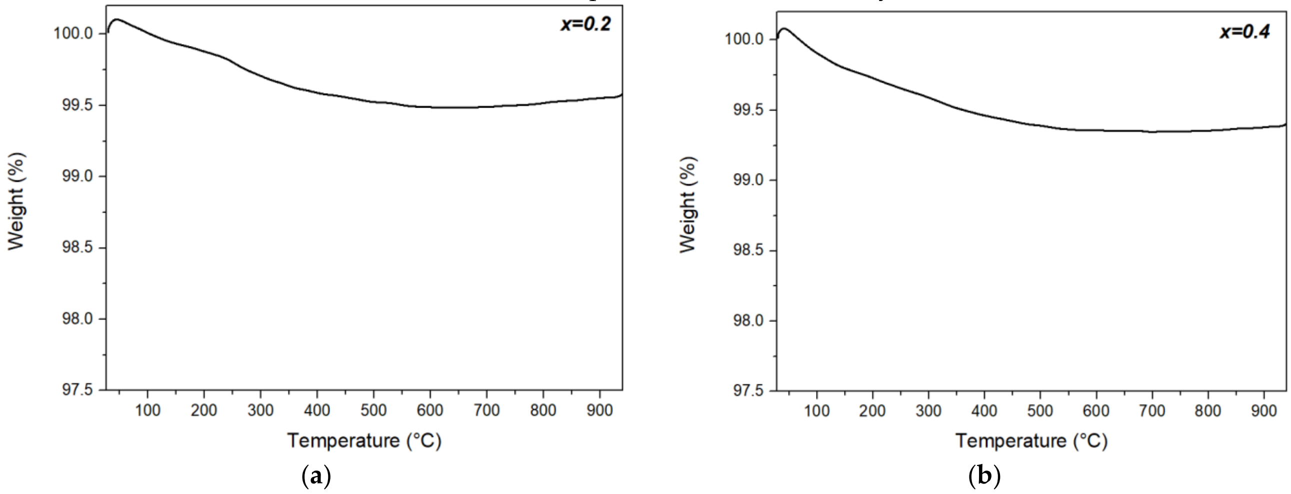

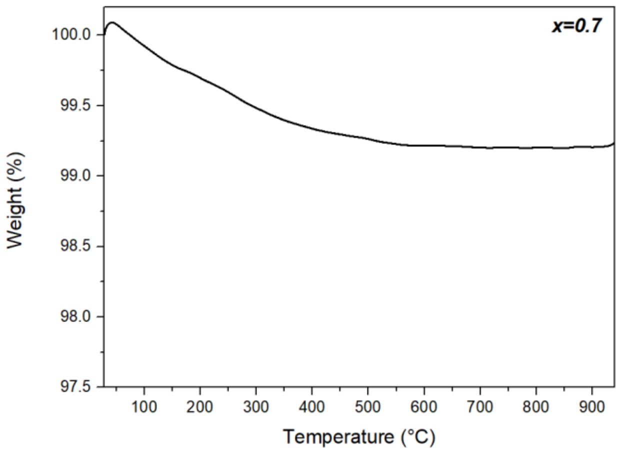

(**c**)

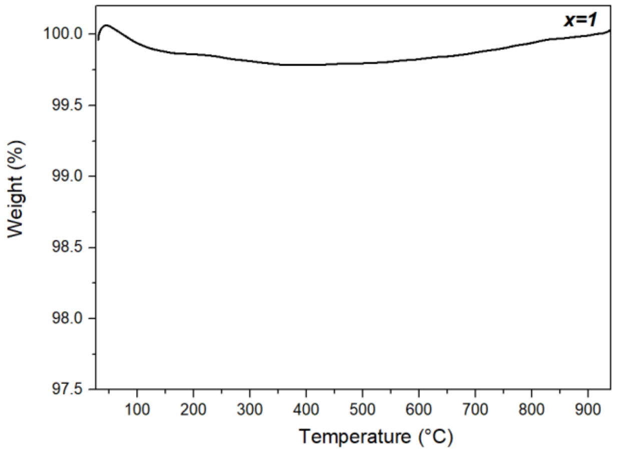

(**d**)

Figure S3. TGA curves of phases in $Al_{2-x}In_xW_3O_{12}$ system: (**a**) x=0.2; (**b**) x=0.4; (**c**) x=0.7 and (**d**) x=1.
